# Adsorption Properties of Hydrocarbon and Fluorocarbon Surfactants Ternary Mixture at the Water-Air Interface

**DOI:** 10.3390/molecules26144313

**Published:** 2021-07-16

**Authors:** Bronisław Jańczuk, Katarzyna Szymczyk, Anna Zdziennicka

**Affiliations:** Department of Interfacial Phenomena, Institute of Chemical Sciences, Faculty of Chemistry, Maria Curie-Skłodowska University in Lublin, Maria Curie-Skłodowska Sq. 3, 20-031 Lublin, Poland; bronislaw.janczuk@poczta.umcs.lublin.pl (B.J.); aniaz@hektor.umcs.lublin.pl (A.Z.)

**Keywords:** adsorption, hydrocarbon surfactant, fluorocarbon surfactant, surface tension, Gibbs free energy of adsorption

## Abstract

Measurements were made of the surface tension of the aqueous solutions of p-(1,1,3,3-tetramethylbutyl) phenoxypoly(ethylene glycols) having 10 oxyethylene groups in the molecule (Triton X-100, TX100) and cetyltrimethylammonium bromide (CTAB) with Zonyl FSN-100 (FC6EO14, FC1) as well as with Zonyl FSO-100 (FC5EO10, FC2) ternary mixtures. The obtained results were compared to those provided by the Fainerman and Miller equation and to the values of the solution surface tension calculated, based on the contribution of a particular surfactant in the mixture to the reduction of water surface tension. The changes of the aqueous solution ternary surfactants mixture surface tension at the constant concentration of TX100 and CTAB mixture at which the water surface tension was reduced to 60 and 50 mN/m as a function of fluorocarbon surfactant concentration, were considered with regard to the composition of the mixed monolayer at the water-air interface. Next, this composition was applied for the calculation of the concentration of the particular surfactants in the monolayer using the Frumkin equation. On the other hand, the Gibbs surface excess concentration was determined only for the fluorocarbon surfactants. The tendency of the particular surfactants to adsorb at the water-air interface was discussed, based on the Gibbs standard free energy of adsorption which was determined using different methods. This energy was also deduced, based on the surfactant tail surface tension and tail-water interface tension.

## 1. Introduction

The ability of surfactants to adsorb at various interfaces and to form micelles in the bulk phase determines their wetting, emulsifying, solubilizing, dispersing, and foaming properties [1]. These properties are the basis for the wide application of surfactants in various industrial fields: pharmacy, medicine, agriculture, and in everyday life [2,3,4,5]. The practical application of surfactants depends on their structure, namely the type and size of the hydrophobic and hydrophilic groups in the surfactant molecules and the types of groups that give these properties [1,2,3,4,5]. Surfactant molecules undergo hydration in the aqueous environment. The hydration of the tail of a surfactant molecule increases the Gibbs free energy of surfactant aqueous solution and hydration of the head causes its decrease. The difference between the increase and decrease of the Gibbs free energy of a surfactant solution determines the strength causing its adsorption at the water−air, water−oil, and solid−water interfaces, as well as the formation of micelles. However, it is not only the surfactants’ tendency to adsorb at the interfaces and the micelle formation that decides about their practical applications. The density, flaccidity, and structure of the created interface layer play a very important role when it comes to the functional properties of surfactants [1].

The density and layer structure of the surfactant interface depend on the components and parameters of their tail and head surface tension [6,7,8]. Unfortunately, it is difficult to find a surfactant that would exhibit all the good properties. Therefore, in practice, mixtures of various surfactant types are used [2,3,4,5]. The application of a surfactant mixture is often associated with the occurrence of a synergistic effect in the reduction of water surface tension. Significant reduction of the water surface tension is necessary, although not sufficient, in the processes in which the wettability of solids plays a priority role. Unfortunately, hydrocarbon surfactants are not able to reduce the water surface tension to the value required for a given process [1,9]. It is known that fluoroalkanes are characterized by lower surface tension than alkanes. Thus, the fluorocarbon surfactants which have a fluoroalkyl chain as a tail reduce the water surface tension to a value lower than that of the hydrocarbons. For this reason, the additives of fluorocarbon surfactants to the hydrocarbon surfactant mixtures are increasingly used in practice [10,11,12,13,14,15,16]. Unfortunately, there are few papers in the literature in which the composition of the adsorption layer at the water-air interface and its density in terms of changes in the water surface tension are discussed [9]. Generally, the composition of the saturated adsorption layer was considered only for a two-component surfactant mixture containing a fluorocarbon surfactant, using Rosen et al. theory [1].

Taking this into account, the aim of our paper was to study the adsorption behavior of the ternary mixtures including two hydrocarbon and one fluorocarbon surfactants at the water-air interface. For these studies Triton X-100 (TX100), cetyltrimethylammonium bromide (CTAB), Zonyl FSN-100 (FC6EO14, FC1) and Zonyl FSO-100 (FC5EO10, FC2) were chosen. These surfactants are very interesting from the practical point of view and can be treated as model surfactants in different mixtures [2,3,4,5,10,11,12,13,14,15,16]. Thus, the aim of the paper was attained by the measurements of the surface tension ($\gamma_{LV}$) of the ternary surfactant mixtures as a function of fluorocarbon surfactant concentration at the constant concentration of CTAB and TX100 mixture corresponding to their aqueous solution surface tension equal to 60 and 50 mN/m [17] and the saturated monolayer at the water−air interface. At these concentrations of the CTAB and TX100 mixture, a significant synergetic effect in the reduction of water surface tension at 293K takes place. The results obtained from surface tension measurements were discussed with regard to the composition of the surface layer, the contribution of the particular surfactants to the reduction of the water surface tension, the concentration of particular components of the mixture in the monolayer and the Gibbs standard free energy of adsorption. The possibility of description and prediction of the surface tension changes as a function of fluorocarbon surfactants concentration was discussed.

## 2. Results and Discussion

### 2.1. Surface Tension of the Aqueous Solution of the Surfactants Ternary Mixture

According to van Oss and Constanzo [18], the surfactants’ surface tension depends on the orientation of their molecules towards the air phase. If the molecules are oriented by tail towards the water phase, then the surface tension of the surfactant is treated as the tail surface tension. In the case of surfactant molecules oriented by head towards the air phase, the surface tension refers to the head. The surface tension of TX100, CTAB, FC1 and FC2 tail results only from the Lifshitz-van der Waals (LW) intermolecular interactions but that of their head results not only from the Lifshitz-van der Waals but also from the acid−base (AB) intermolecular interactions [9,19,20]. The water surface tension results also from the LW and AB intermolecular interactions [21,22]. As the surfactant molecules in the monolayer at the water-air interface are oriented by their tail towards the air phase, thus the values of the surface tension of the aqueous solution of the mixture including two hydrocarbon and one fluorocarbon surfactants depend on those of the water and surfactant tail surface tension. The surface tension of water at 293 K is equal to 72.8 mN/m and the LW and AB components of this tension are equal to 26.85 and 45.95 mN/m, respectively [22]. In turn, the surface tension of TX100, CTAB, FC1 and FC2 tails is equal to 22.0, 27.00 [19], 11.91, and 9.89 mN/m, respectively [23]. Thus, theoretically the surface tension of the aqueous solution of CTAB should be changed from 72.8 to 27.0 mN/m, TX100 from 72.8 to 22.0 mN/m, FC1 from 72.8 to 11.91 mN/m and FC2 from 72.8 to 9.89 mN/m. In fact, the minimum surface tension of the aqueous solutions of these single surfactants was equal to 37.8, 33.8 [7], 23.7 and 19.4 mN/m [20].

**Figure 1:**
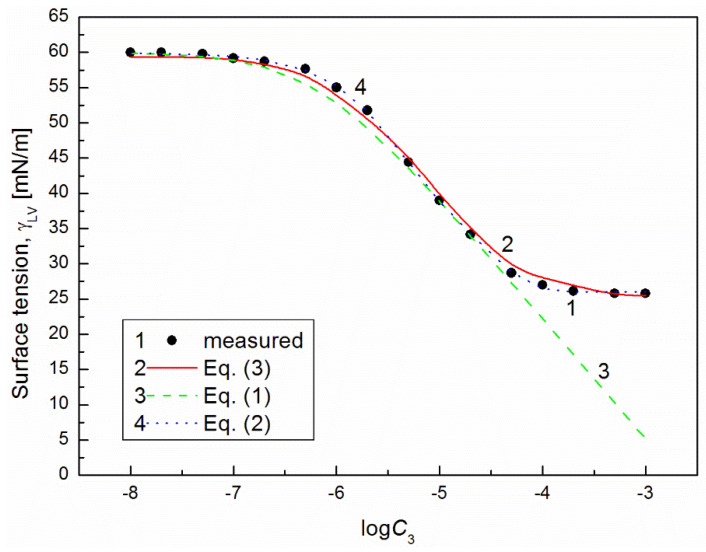
A plot of the surface tension ($\gamma_{LV}$) of the aqueous solutions of CTAB + TX100 + FC1 mixture at the constant concentration of the binary mixture CTAB + TX100 ($C_{12}$) and $\gamma_{LV}$ = 60 mN/m (m1 (60)) vs. the logarithm of FC1 concentration ($logC_{3}$). Points 1 correspond to the measured values, curves 2, 3 and 4 correspond to the values calculated from the Fainerman and Miller (Equation (3)), the Szyszkowski equation (Equation (1)) and the second order exponential function (Equation (2)), respectively.

**Figure 2:**
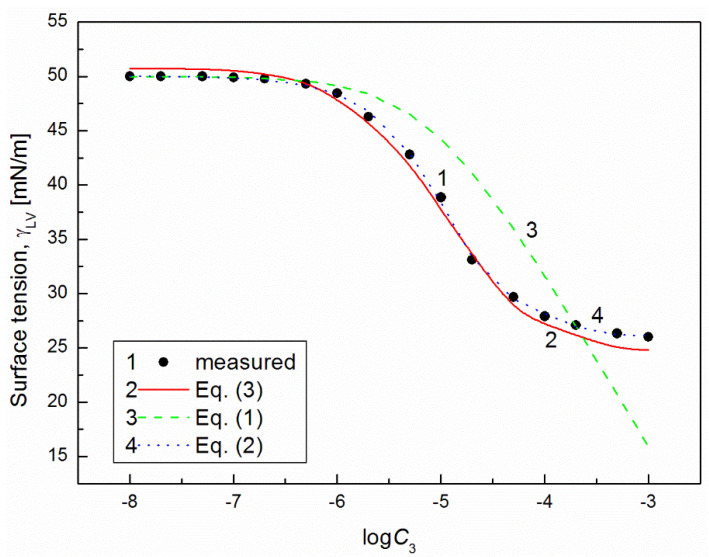
A plot of the surface tension ($\gamma_{LV}$) of the aqueous solutions of CTAB + TX100 + FC1 mixture at the constant concentration of the binary mixture CTAB + TX100 ($C_{12}$) and $\gamma_{LV}$ = 50 mN/m (m1 (50)) vs. the logarithm of FC1 concentration ($logC_{3}$). Points 1 correspond to the measured values, curves 2, 3 and 4 correspond the values calculated from the Fainerman and Miller (Equation (3)), the Szyszkowski equation (Equation (1)) and the second order exponential function (Equation (2)), respectively.

**Figure 3:**
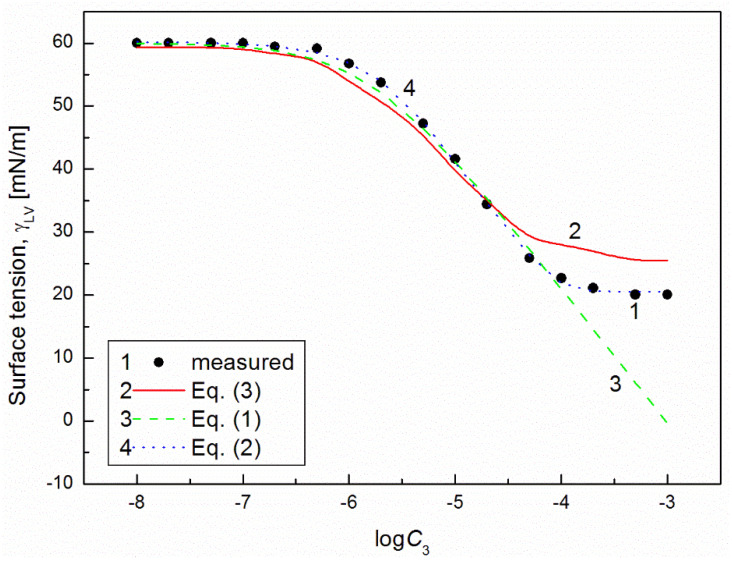
A plot of the surface tension ($\gamma_{LV}$) of the aqueous solutions of CTAB + TX100 + FC2 mixture at the constant concentration of the binary mixture CTAB + TX100 ($C_{12}$) and $\gamma_{LV}$ = 60 mN/m (m2 (60)) vs. the logarithm of FC2 concentration ($logC_{3}$). Points 1 correspond to the measured values, curves 2, 3 and 4 correspond the values calculated from the Fainerman and Miller (Equation (3)), the Szyszkowski equation (Equation (1)) and the second order exponential function (Equation (2)), respectively.

**Figure 4:**
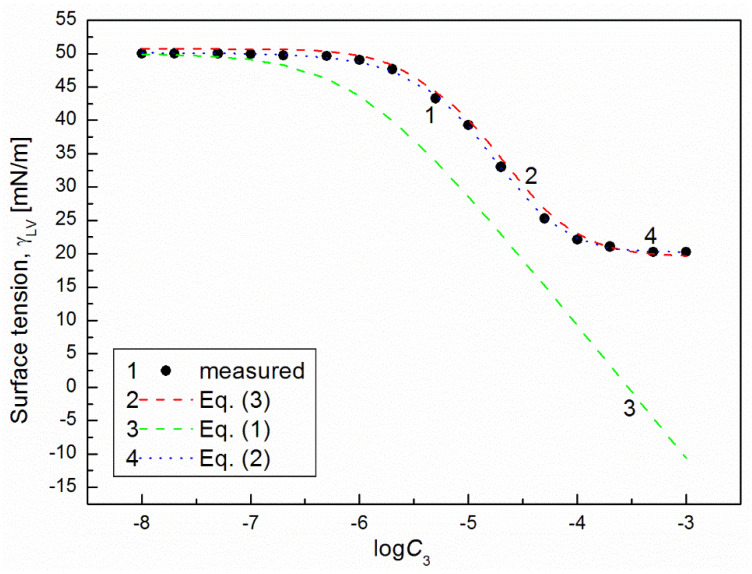
A plot of the surface tension ($\gamma_{LV}$) of the aqueous solutions of CTAB + TX100 + FC2 mixture at the constant concentration of the binary mixture CTAB + TX100 ($C_{12}$) and $\gamma_{LV}$ = 50 mN/m (m2 (50)) vs. the logarithm of FC2 concentration ($logC_{3}$). Points 1 correspond to the measured values, curves 2, 3 and 4 correspond the values calculated from the Fainerman and Miller (Equation (3)), the Szyszkowski equation (Equation (1)) and the second order exponential function (Equation (2)), respectively.

It is interesting that the differences between the minimal surface tension of the aqueous solution of studied surfactants and their tail surface tension were almost the same, being 10.8, 11.8, 9.51 and 11.71 mN/m [7,20], respectively. From the comparison of the minimal values of the surface tension of the aqueous solutions of particular surfactants, it resulted that the addition of the fluorocarbon surfactant (FC) to the hydrocarbon ones should decrease the surface tension of the aqueous solution of hydrocarbon surfactants mixture considerably. The isotherm of the surface tension of the aqueous solution of the ternary mixture at the constant concentration of TX100 and CTAB confirms this statement (Figure 1, Figure 2, Figure 3 and Figure 4). The isotherms of $\gamma_{LV}$ for the ternary mixtures are similar to those of single surfactants [7,20]. Both a rectilinear segment and an inflection point were observed on them. The linear dependence between $\gamma_{LV}$ and the logarithm of the fluorocarbon surfactant concentration suggests that there is the constant Gibbs surface excess concentration and the inflection point indicates that the formation of the mixed micelles takes place.

As mentioned above, the concentration of the TX100 and CTAB mixture corresponded to the saturated mixed monolayer at the water−air interface [17]. However, the addition of the fluorocarbon surfactants to this mixture decreased its surface tension (Figure 1, Figure 2, Figure 3 and Figure 4). The difference between the LW component of water surface tension and the surface tension of CTAB and TX100 was smaller than that between LW of water and the tail of fluorocarbon surfactants. Thus at the adsorption of hydrocarbon surfactants, the decrease of the water surface tension results rather from the decrease of AB components of its surface tension than from the LW one.

In the case of fluorocarbon surfactants both LW and AB components of the water surface tension can be reduced at their adsorption at the water−air interface. The problem is why the reduction of the water surface tension increases in the range of the fluorocarbon surfactant concentration corresponding to that of the saturated mixed monolayer at the water−air interface. Commonly, it is assumed that this phenomenon results from the micelle formation [1]. However, the process of the micellization does not influence the structure of the water in the interface region. It is possible that with an increase of surfactant concentration in the range corresponding to the constant Gibbs surface excess concentration at the water-air interface, the concentration gradient of the surfactants increases and the concentration in the interface region increases slightly. This causes the separation of the water molecules which diminishes the possibility of hydrogen bond formation and as a result the surface tension of the solution decreases. In fact, in the studied systems, the replacement of the CTAB and TX100 molecules by the fluorocarbon ones took place.

In the case of single aqueous solutions of CTAB, TX100, FC1 and FC2, it was possible to describe the changes of $\gamma_{LV}$ as a function of its concentration in the range from zero to CMC by the Szyszkowski equation [1,24]. From the theoretical point of view it was interesting to consider whether the changes of the $\gamma_{LV}$ for the aqueous solution of the ternary mixtures of the surfactants as a function of concentration could be described by this equation, which in the case of our systems has the following form [1,24]:

(1) $$\gamma_{0}-\gamma_{LV}=\pi =-RT\Gamma^{max}ln\left(\frac{C_{3}}{a}+1\right),$$

where: $\gamma_{0}$ is the surface tension of the aqueous solution of CTAB and TX100 mixtures equal 60 or 50 mN/m, $C_{3}$ is the concentration of the fluorocarbon surfactant, $\Gamma^{max}$ is the maximal surface concentration of FC close to the Gibbs surface excess concentration, *T* is the temperature and *R* is the gas constant. $\Gamma^{max}$ can be determined from the slope of the linear dependence between $\gamma_{LV}$ and log$C_{3}$ and a is the constant being a function of the Gibbs free energy of adsorption.

It appears that it is possible to choose an appropriate value of a for which the values of the solution surface tension obtained from Equation (1) for the aqueous solution of the ternary mixture at the constant CTAB and TX100 concentration at $\gamma_{LV}$ equal to 60 mN/m are close to those measured in the FC concentration range from zero to CMC (Figure 1 and Figure 3). In the case of the constant concentration of CTAB and TX100 at $\gamma_{LV}$ equal to 50 mN/m there are significant differences between the measured and calculated values of the aqueous solution surface tension of the ternary mixture (Figure 2 and Figure 4). It should be noted that the Szyszkowski equation was proposed based on the surface tension changes of the aqueous solution of a single surface active agent as a function of its concentration. In such a case, the concentration of the surface active agents in the monolayer at the W-A interface increases as a function of its concentration in the bulk phase to a maximal value. In the case of the surfactants mixture, the changes of the concentration of the given surfactant in the mixed monolayer at the W-A interface as a function of surfactants mixture concentration in the bulk phase can be different from the changes of this surfactant concentration in the absence of other surfactants. Maybe, for this reason, it is difficult to describe exactly by equation the changes of $\gamma_{LV}$ as a function of FC concentration at the different values of the constant concentration of the CTAB and TX100 mixture by means of Equation (1). However, in contrast to Equation (1) for both the constant concentration of the CTAB and TX100 mixture the changes of $\gamma_{LV}$ as a function of $C_{3}$ can be described with good accuracy in the whole range of $C_{3}$ using the exponential function of the second order which can be expressed as:

(2) $$\gamma_{LV}=y_{o}+A_{1}exp\left(-\frac{C_{3}}{t_{1}}\right)+A_{2}exp\left(-\frac{C_{3}}{t_{2}}\right),$$

where: $y_{o}$, $A_{1}$, $A_{2}$, $t_{1}$ and $t_{2}$ are the constants which are difficult to express as a function of some physicochemical properties of the solution. These constants depend on the type of FC and the value of the surface tension of the solution of CTAB and TX100 mixture (Table 1).

Fainerman and Miller derived the equation on the basis of which it is possible to predict the surface tension of the aqueous solution of the mixture of two hydrocarbon surfactants, taking into account the surface layer pressure of the single surfactants solution [25,26]. According to thermodynamic rules, this equation can be applied for the aqueous solutions of multicomponent mixtures of surfactants. Thus for the ternary surfactants mixture, the Fainerman and Miller equation [25,26] can be expressed in the form:

(3) $$exp\Pi^{*}=exp\Pi_{1}^{*}+exp\Pi_{2}^{*}+exp\Pi_{3}^{*}-1,$$

where: $\Pi^{*}=\Pi \omega /RT$, $\Pi_{1}^{*}=\Pi_{1}\omega_{1}/RT$, $\Pi_{2}^{*}=\Pi_{2}\omega_{2}/RT$, and $\Pi_{3}^{*}=\Pi_{3}\omega_{3}/RT$ are the dimensionless surface pressures of the mixture and individual solutions of surfactants 1 (TX100), 2 (CTAB) and 3 (FC1 or FC2), and $\omega_{1}$, $\omega_{2}$, $\omega_{3}$ and ω are the molar surface areas of the surfactants 1, 2 and 3 and their mixture, and $\Pi_{1}$, $\Pi_{2}$ and $\Pi_{3}$, are the differences between the surface tension of the solvent and solution of the surface active agents 1, 2 and 3, respectively.

To solve Equation (3) against the surface tension of the aqueous solution of CTAB, TX100 and FC1 or FC2 mixtures, it is necessary to determine their molar surface area. This results from the fact that the surface molar areas of CTAB, TX100 and FC are different and the composition of ternary mixtures in the bulk phase changes with the FC concentration. It seems that the molar area of this mixture should depend on the fraction of the particular surfactants to the mixture in the reduction of the water surface tension. Thus the ω can be expressed by the following equation:

(4) $$\omega =X_{1}\omega_{1}+X_{2}\omega_{2}+X_{3}\omega_{3},$$

where: $X_{1}$, $X_{2}$ and $X_{3}$ are the fractions of contribution of TX100, CTAB and FC to the reduction of water surface tension. The values of $X_{1}$, $X_{2}$ and $X_{3}$ can be determined on the basis of $\Pi_{1}$, $\Pi_{2}$ and $\Pi_{3}$ values $\left(X_{1}=\frac{\Pi_{1}}{\Pi_{1}+\Pi_{2}+\Pi_{3}},\;X_{2}=\frac{\Pi_{2}}{\Pi_{1}+\Pi_{2}+\Pi_{3}},X_{3}=\frac{\Pi_{3}}{\Pi_{1}+\Pi_{2}+\Pi_{3}}\right)$. For the $\gamma_{LV}$ calculations from Equation (3) the values of $\omega_{1}$, $\omega_{2}$, $\omega_{3}$ as well as $\Pi_{1}$, $\Pi_{2}$ and $\Pi_{3}$ were taken from the literature [7,9,20]. It appears that the values of $\gamma_{LV}$ calculated from Equation (3) are close to those measured in the whole range of FC concentration (Figure 1, Figure 2, Figure 3 and Figure 4). Thus we can conclude that the Fainerman and Miller equation can be used not only for the ideal solutions of binary mixture of surfactants but also for the ternary one.

### 2.2. Composition of Mixed Monolayer at the Water-Air Interface

**Figure 5:**
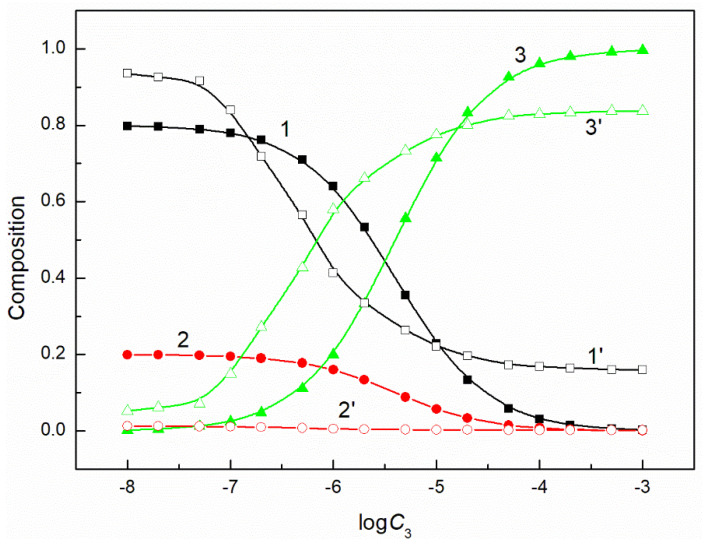
A plot of the composition of the ternary mixture including CTAB, TX100 and FC1 in the monolayer (curves 1–3) and in the bulk phase (curves 1’–3’) at the constant $C_{12}$ and $\gamma_{LV}$ = 60 mN/m vs. the logarithm of the FC1 concentration ($logC_{3}$). Curves 1, 1’ correspond to CTAB, curves 2, 2’ correspond to TX100 and curves 3, 3’ to FC1.

**Figure 6:**
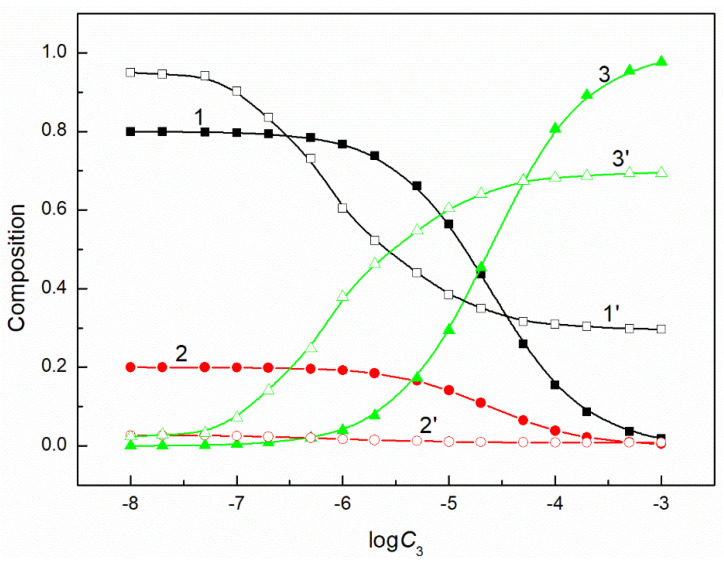
A plot of the composition of the ternary mixture including CTAB, TX100 and FC1 in the monolayer (curves 1–3) and in the bulk phase (curves 1’–3’) at the constant $C_{12}$ and $\gamma_{LV}$ = 50 mN/m vs. the logarithm of the FC1 concentration ($logC_{3}$). Curves 1, 1’ correspond to CTAB, curves 2, 2’ correspond to TX100 and curves 3–3’ to FC1.

**Figure 7:**
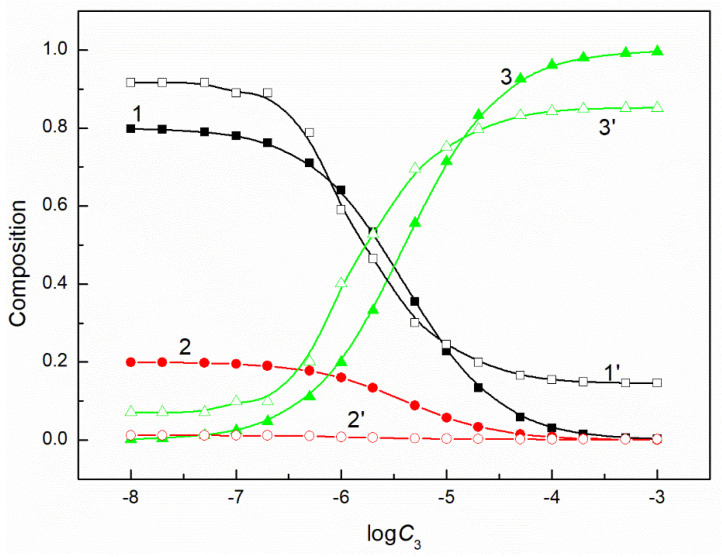
A plot of the composition of the ternary mixture including CTAB, TX100 and FC2 in the monolayer (curves 1–3) and in the bulk phase (curves 1’–3’) at the constant $C_{12}$ and $\gamma_{LV}$ = 60 mN/m vs. the logarithm of the FC2 concentration ($logC_{3}$). Curves 1, 1’ correspond to CTAB, curves 2, 2’ correspond to TX100 and curves 3–3’ to FC2.

**Figure 8:**
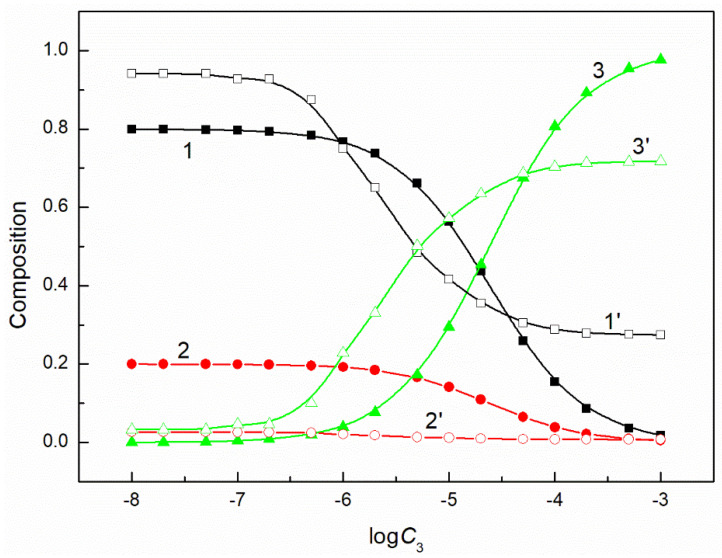
A plot of the composition of the ternary mixture including CTAB, TX100 and FC2 in the monolayer (curves 1–3) and in the bulk phase (curves 1’–3’) at the constant $C_{12}$ and $\gamma_{LV}$ = 50 mN/m vs. the logarithm of the FC2 concentration ($logC_{3}$). Curves 1, 1’ correspond to CTAB, curves 2, 2’ correspond to TX100 and curves 3–3’ to FC2.

The composition of the mixed surface layer at the water−air interface influences the reduction of the water surface tension. In some cases, the synergetic effect takes place in this reduction. It was mentioned above that the fraction of the given component of the mixed layer pressure can be determined on the basis of its individual layer pressure. It can be assumed that this fraction is related to the mole fraction of a given component in the mixed surface layer. If so, to explain the differences in the adsorption activity of the mixture components, this fraction can be compared with the composition of the surfactants mixture in the bulk phase. Our studied systems contain a constant concentration of the mixture of CTAB and TX100 and the concentration of FC changes. Thus, FC molecules remove those of CTAB and TX100 from the mixed monolayer at the water−air interface. However, the changes of the composition of the ternary mixture layer as a function of the FC concentration are not proportional to those of a composition of a given mixture in the bulk phase (Figure 5, Figure 6, Figure 7 and Figure 8). The adsorption activity of the components of the ternary mixture increases from CTAB to FC [7,27]. This indicates that the concentration of FC in the mixed monolayer should increase compared to the bulk phase and those of CTAB and TX100 should decrease. It should be noted that this is the case, but not in the whole range of FC concentration in the bulk phase. At a low concentration of FC and higher than the CMC of the mixture, the TX100 concentration in the mixed monolayer is larger than in the bulk phase. On the other hand, the concentration of FC in the mixed layer is much higher than in the bulk phase, but this takes place in the concentration range from zero to CMC. Indeed, in the whole concentration range of FC the CTAB concentration in the monolayer is considerably lower than in the bulk phase.

Considering the adsorption activity of surfactants, it is surprising that at a concentration of the mixture in the bulk phase higher than CMC, the FC concentration in the surface layer is lower than in the bulk phase. This may result from the fact that FC have a greater tendency to form micelles than to adsorb at the water-air interface. In the case of TX100 the increase of its concentration in the surface layer in comparison to the bulk phase can result from the greater removal of CTAB ions from the surface layer than of TX100 molecules by FC ones.

To examine the credibility of the values of the molar fraction of particular components in the ternary mixed monolayer at the water−air interface determined based on the contribution of the particular surfactant to the reduction of water surface tension, the method proposed by Rosen and Hua [28] was applied. In this method the nonideal solution theory was used. However, the equation obtained based on this theory deals with the binary mixtures of the surfactants. This equation can be used for the calculation of the molar fraction of the components of a ternary mixture [24]. In such a case, the binary mixture (CTAB + TX100) is treated as one surfactant. For this case, the Rosen and Hua equations has the form [1,28]:

(5) $$\frac{{\left(X_{12}^{s}\right)}^{2}ln\left(\alpha_{12}C_{123}/X_{12}^{s}C_{12}\right)}{{\left(1-X_{12}^{s}\right)}^{2}ln\left[\left(1-\alpha_{12}\right)C_{123}/\left(1-X_{12}^{s}\right)C_{2}\right]}=1$$

where: $\alpha_{12}$ is the mole fraction of CTAB+TX100 in the bulk phase, $C_{12}$—the concentration of the TX100 and CTAB binary mixture, $C_{3}$—the concentration of FC, $C_{123}$—the concentration of the ternary mixture of CTAB + TX100 + FC at the same value of surface tension of their aqueous solution, $X_{12}^{s}$—the sum of the CTAB ($X_{1}$) and TX100 ($X_{2}$) mole fractions. The FC mole fraction, $X_{3}^{s},$ is equal to: $X_{3}^{s}=1-X_{12}^{s}$.

**Figure 9:**
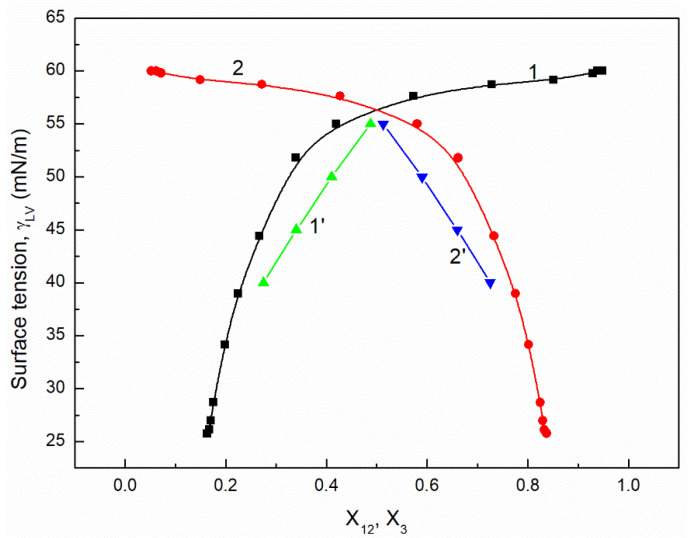
A plot of the surface tension ($\gamma_{LV}$) of the aqueous solutions of CTAB + TX100 + FC1 mixture at the constant concentration of the binary mixture CTAB + TX100 ($C_{12}$) and $\gamma_{LV}$ = 60 mN/m (m1 (60) vs. the composition of the ternary mixture in the monolayer ($X_{12}$ and $X_{3}$) determined based on the contribution of the particular surfactant in the mixture to the reduction of the water surface tension (curves 1 and 2) and calculated from Equation (5) (curves 1’ and 2’). $X_{12}$ corresponds to the sum of the CTAB ($X_{1}$) and TX100 ($X_{2}$) fractions and $X_{3}$ corresponds to the FC1 fraction.

**Figure 10:**
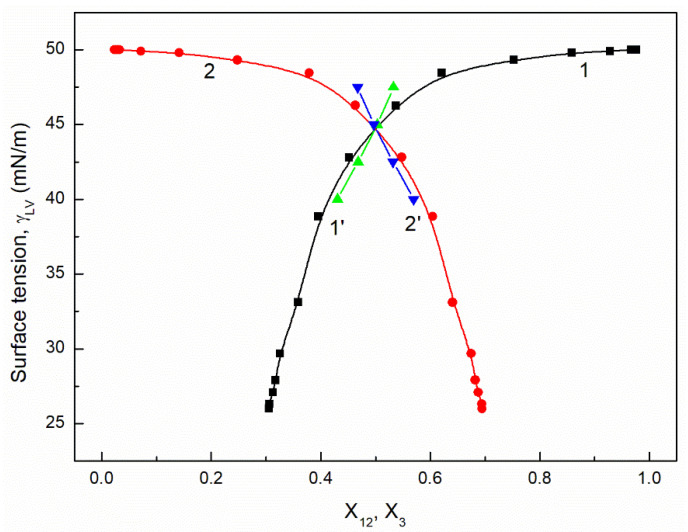
A plot of the surface tension ($\gamma_{LV}$) of the aqueous solutions of CTAB + TX100 + FC1 mixture at the constant concentration of the binary mixture CTAB + TX100 ($C_{12}$) and $\gamma_{LV}$ = 50 mN/m (m1 (50)) vs. the composition of the ternary mixture in the monolayer ($X_{12}$ and $X_{3}$) determined based on the contribution of the particular surfactant in the mixture to the reduction of the water surface tension (curves 1 and 2) and calculated from Equation (5) (curves 1’ and 2’). $X_{12}$ corresponds to the sum of the CTAB ($X_{1}$) and TX100 ($X_{2}$) fractions and $X_{3}$ corresponds to the FC1 fraction.

**Figure 11:**
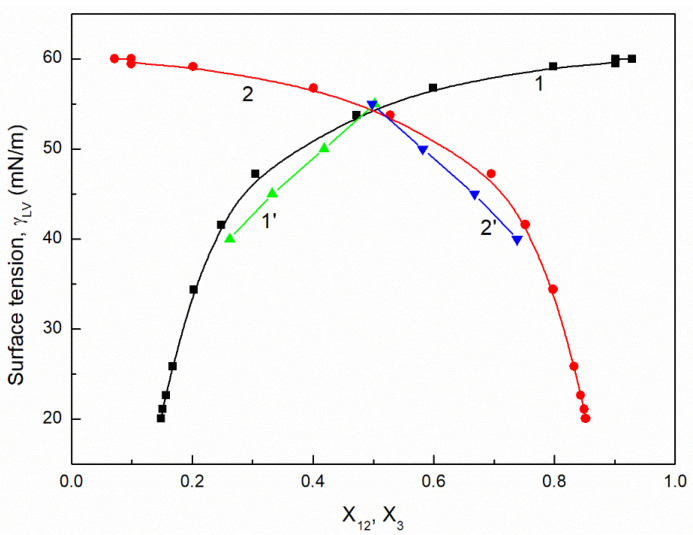
A plot of the surface tension ($\gamma_{LV}$) of the aqueous solutions of CTAB + TX100 + FC2 mixture at the constant concentration of the binary mixture CTAB + TX100 ($C_{12}$) and $\gamma_{LV}$ = 60 mN/m (m2 (60)) vs. the composition of the ternary mixture in the monolayer ($X_{12}$ and $X_{3}$) determined based on the contribution of the particular surfactant in the mixture to the reduction of the water surface tension (curves 1 and 2) and calculated from Equation (5) (curves 1’ and 2’). $X_{12}$ corresponds to the sum of the CTAB ($X_{1}$) and TX100 ($X_{2}$) fractions and $X_{3}$ corresponds to the FC2 fraction.

**Figure 12:**
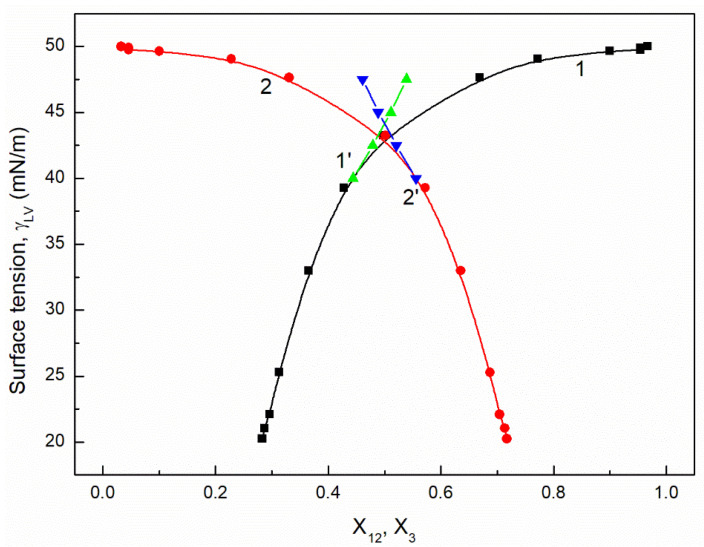
A plot of the surface tension ($\gamma_{LV}$) of the aqueous solutions of CTAB + TX100 + FC2 mixture at the constant concentration of the binary mixture CTAB + TX100 ($C_{12}$) and $\gamma_{LV}$ = 50 mN/m (m2 (50)) vs. the composition of the ternary mixture in the monolayer ($X_{12}$ and $X_{3}$) determined based on the contribution of the particular surfactant in the mixture to the reduction of the water surface tension (curves 1 and 2) and calculated from Equation (5) (curves 1’ and 2’). $X_{12}$ corresponds to the sum of the CTAB ($X_{1}$) and TX100 ($X_{2}$) fractions and $X_{3}$ corresponds to the FC2 fraction.

It proves that the mole fractions of the CTAB+TX100 and FC calculated from Equation (5) are close to those calculated based on the contribution of the particular surfactant to the reduction of water surface tension (Figure 9, Figure 10, Figure 11 and Figure 12). This indicates that, based on the surfactant pressure of its individual monolayers, it is possible to predict the composition of the multicomponent mixed monolayer at the water−air interface.

From the mole fraction of the surfactant in the monolayer, it is possible to calculate the parameter of intermolecular interaction ($\beta^{\sigma }$) in this layer using the following equation [1,28]:

(6) $$\beta^{\sigma }=\frac{ln\left(\alpha_{12}C_{123}/X_{12}^{s}C_{12}\right)}{{\left(1-X_{12}^{s}\right)}^{2}},$$

The values of the intermolecular interaction- parameter calculated from Equation (6) are negative (Table 2). According to Rosen [1] this means that there is a synergetic effect on the reduction of the water surface tension by the ternary mixture CTAB, TX100 and FC.

The synergetic effect may be related to the increase in the density of the mixed monolayer compared to that of individual surfactants contained in the mixture. The increase in the density may be related to the Gibbs excess free energy of mixing $G_{mix}^{E}$. This energy can be determined from the following equation [1,29]:

(7) $$G_{mix}^{E}=RT\left(X_{12}^{s}lnf_{12\;}+X_{3}^{s}lnf_{3}\right),$$

where: $f_{12}$ and $f_{3}$ are the activity coefficients of the CTAB and TX100 mixture and FC surfactant in the surface layer. These coefficients can be expressed as:

(8) $$lnf_{12}=\beta^{\sigma }{\left(1-X_{12}^{s}\right)}^{2},$$

(9) $$lnf_{3}=\beta^{\sigma }{\left(X_{12}^{s}\right)}^{2},$$

In all cases the values of $G_{mix}^{E}$ calculated from Equation (7) (Table 2) are negative. This indicates that spontaneous mixing of the TX100 and CTAB mixture with FC in the surface layer of the ternary mixtures takes place. This is probably due to the reduction of repulsive interactions between the hydrophilic groups of TX100 and CTAB by the FC molecules. It is also possible that the hydrophobic interactions between the FC molecules and those of CTAB and TX100 are greater than between the TX100 and CTAB ones.

### 2.3. Concentration of CTAB, TX100 and FC Surfactants in the Mixed Monolayer at the Water-Air Interface

Based on the surface tension values it is possible to determine the concentration of the particular surfactants at the water-air interface. In the literature, one can find many methods used for this determination. Among them the Gibbs and Frumkin isotherms of adsorption can be used [1,24]. In the case of the Gibbs equation only the surface excess concentration of surfactants can be found. On the other hand, the surfactants concentration in the bulk phase is considerably lower than in the adsorption monolayer. Thus, this Gibbs surface excess concentration of surfactants can be treated at the approximation as total one. However, for the studied systems the Gibbs isotherm of adsorption equation for determination of only FC concentration can be used. For FC, the Gibbs isotherm equation can be expressed in the form [1,24]:

(10) $$\Gamma_{3}=-\frac{C_{3}}{RT}{\left(\frac{\partial \gamma_{LV}}{\partial C_{3}}\right)}_{T,C_{12}}=-\frac{1}{2.303RT}{\left(\frac{\partial \gamma_{LV}}{\partial logC_{3}}\right)}_{T,C_{12}},$$

where: $\Gamma_{3}$ is the Gibbs surface excess concentration of FC.

**Figure 13:**
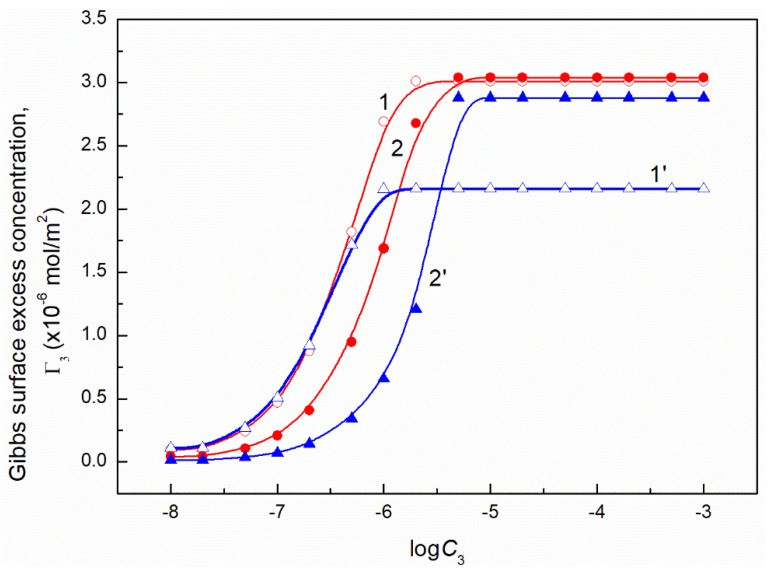
A plot of the Gibbs surface excess concentration of FC1 ($\Gamma_{3}$) calculated from Equation (9) based on the measured surface tension of the ternary mixture at the constant concentration of the CTAB + TX100 at $\gamma_{LV}$ equal 60 mN/m (curve 1) and 50 mN/m (curve 2) and on the contribution of FC1 to the reduction of water surface tension (curve 1’corresponds to $\gamma_{LV}$ = 60 mN/m and curve 2’corresponds to $\gamma_{LV}$ = 50 mN/m) vs. the logarithm of the FC1 concentration ($logC_{3}$).

**Figure 14:**
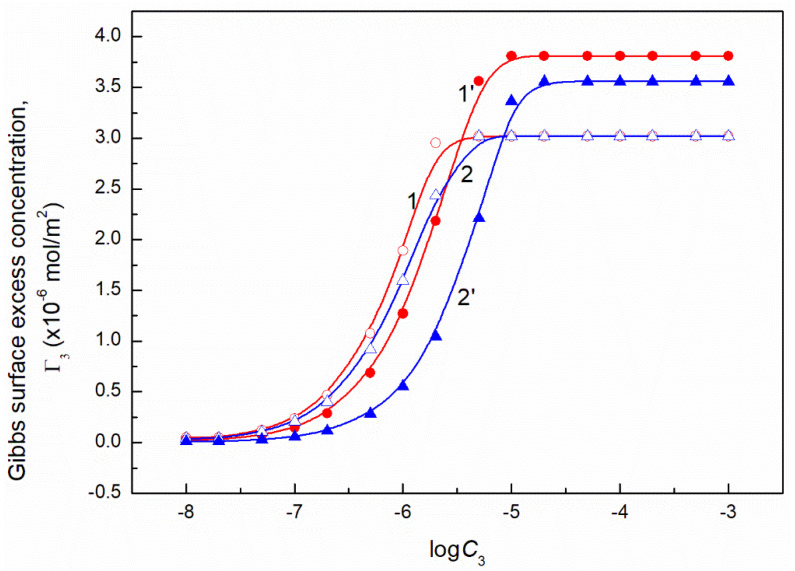
A plot of the Gibbs surface excess concentration of FC1 ($\Gamma_{3}$) calculated from Equation (9) based on the measured surface tension of the ternary mixture at the constant concentration of the CTAB + TX100 at $\gamma_{LV}$ equal 60 mN/m (curve 1) and 50 mN/m (curve 2) and on the contribution of FC2 to the reduction of water surface tension (curve 1’corresponds to $\gamma_{LV}$ = 60 mN/m and curve 2’ corresponds to $\gamma_{LV}$ = 50 mN/m) vs. the logarithm of the FC2 concentration ($logC_{3}$).

It should be remembered that Equation (10) is derived on the assumption that the activity coefficient of the surfactant is close to unity and the number of the water moles in 1 dm^3^ is almost constant in the studied surfactant concentration range. It is equal to 55.41 at 293 K. To solve Equation (10) the values of $\frac{\partial \gamma_{LV}}{\partial C_{3}}$ or $\frac{\partial \gamma_{LV}}{\partial logC_{3}}$ must be known. As mentioned above the dependence between the surface tension of the aqueous solution of ternary surfactants mixture and FC concentration can be described by the exponential function of the second order. Thus it was possible to determine $\frac{\partial \gamma_{LV}}{\partial C_{3}}$. However, at the FC concentration at which the linear dependence was observed (Figure 1, Figure 2, Figure 3 and Figure 4), the more real values of $\Gamma_{3}$ were obtained using the $\frac{\partial \gamma_{LV}}{\partial logC_{3}}$ values (Figure 13 and Figure 14).

The values of $\Gamma_{3}$ calculated from Equation (10) (Figure 13 and Figure 14) indicate that the adsorption of FC increases as a function of its concentration. Seemingly, this seems to be a normal phenomenon. However, we must remember that the FC is added to TX100 and CTAB mixture at the concentration corresponding to the saturated monolayer of binary mixture at the water−air interface. This means that the FC molecules increase the density of the surface monolayer or remove CTAB or TX100 molecules and both these two processes take place at the same time. The increase of the FC concentration in the surface monolayer at the concentration of CTAB and TX100 mixture corresponding to the surface tension of their aqueous solution equal to 50 mN/m is smaller than at the surface tension equal to 60 mN/m. This suggests that not only the intermolecular interactions in the surface region but also in the bulk phase between the FC molecules and CTAB and TX100 influence the adsorption of FC. This is possible due to the strong hydrophilic interactions between TX100 and FC molecules which result in the formation of dimmers of these surfactants in the bulk phase with different hydrophilic group conformations. In these cases, changes of the hydration degree of the tail and head of surfactants can take place, affecting their tendency to adsorb at the water-air interface.

The maximal values of $\Gamma_{3}$ for FC1 and FC2 are smaller than those obtained in the absence of TX100 and CTAB [27]. These values at $C_{12}$ corresponding to the surface tension equal to 50 mN/m are smaller for each FC than those at the surface tension equal to 60 mN/m (Figure 13 and Figure 14). The maximal surface excess concentration of FC1 is smaller than that of FC2 in the absence of other surfactants [27] which probably results from smaller ratio of hydrophobic groups to the oxyethylene units. This can also result from the lower difference between the surface tension of fluorocarbon being a chain of FC1 and the FC1−water interface tension [20] than in the case of FC2. Probably for this reason the maximal surface excess concentration for FC2 is higher than that of FC1 in the mixed surface layer at the water−air interface. It should be mentioned that the $\Gamma_{3}$ values calculated from Equation (9) for both FC surfactants satisfy the linear Langmuir and Gu and Zhu equations which have the forms [1,24,30,31]:

(11) $$\frac{C_{3}}{\Gamma_{3}}=\frac{C_{3}}{\Gamma_{3}^{max}}+\frac{a}{\Gamma_{3}^{max}},$$

and

(12) $$log\frac{\Gamma_{3}}{\Gamma_{3}^{\infty }-\Gamma_{3}}=logK+nlogC_{3},$$

where: $\Gamma_{3}^{\infty }$ is the limiting surface excess concentration of FC, $\Gamma_{3}^{max}$ is the maximal Gibbs surface excess concentration of FC, *n* is the aggregation number and *K* is the equilibrium constant associated with the aggregation process. For all studied systems Equation (12) has the linear form at the slope equal to 1. In such a case, K=1/a. The a constant is connected with the Gibbs free energy of adsorption. It should be mentioned that for both FC1 and FC2 a constant in Equation (11) is close to that in Equation (12) as well as to that in Equation (1).

Unfortunately, using the Gibbs isotherm equation it is impossible to determine the changes of the TX100 and CTAB concentration in the mixed layer as a function of $C_{3}$. However, it can be done using the Frumkin equation, which can be expressed in the following form [1,24]:

(13) $$\pi_{1}=-RT\Gamma_{i}^{max}ln\left(1-\frac{\Gamma_{i}}{\Gamma_{i}^{max}}\right),$$

where: $\pi_{i}$ is the contribution of *i* component in the monolayer to reduction of the water surface tension. The main problem for solving Equation (13) against $\Gamma_{i}$ is to find the $\pi_{i}$ values for each component of the mixed ternary surface monolayer.

**Figure 15:**
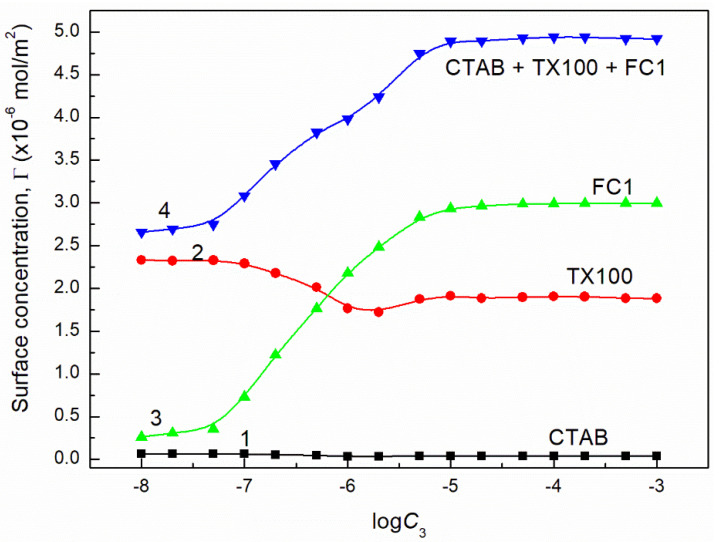
A plot of the concentration of CTAB (curve 1), TX100 (curve 2), FC1 (curve 3) and their sum (curve 4) in the mixed monolayer at the water-air interface formed by the CTAB + TX100 + FC1 mixture at the constant concentration of the binary CTAB + TX100 mixture ($C_{12}$) and $\gamma_{LV}$ = 60 mN/m as well as calculated from Equation (12) based the contribution of the given surfactant to the reduction of water surface tension vs. the logarithm of the FC1 concentration ($logC_{3}$).

**Figure 16:**
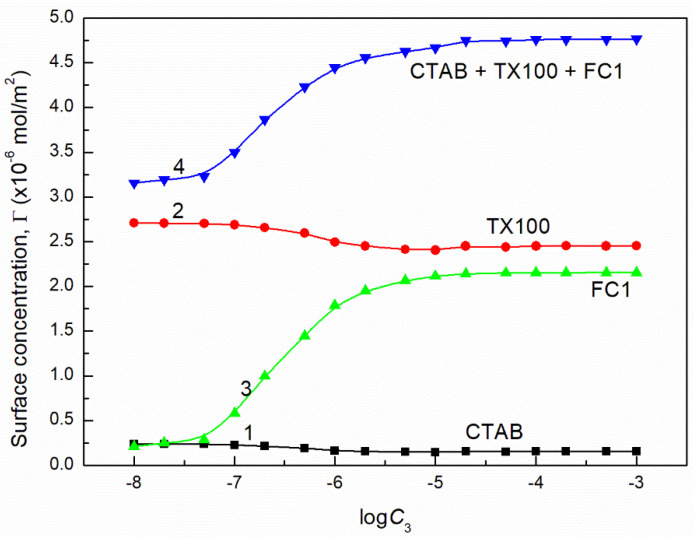
A plot of the concentration of CTAB (curve 1), TX100 (curve 2), FC1 (curve 3) and their sum (curve 4) in the mixed monolayer at the water-air interface formed by the CTAB + TX100 + FC1 mixture at the constant concentration of the binary CTAB + TX100 mixture ($C_{12}$) and $\gamma_{LV}$ = 50 mN/m as well as calculated from Equation (12) based on the contribution of the given surfactant to the reduction of water surface tension vs. the logarithm of the FC1 concentration ($logC_{3}$).

**Figure 17:**
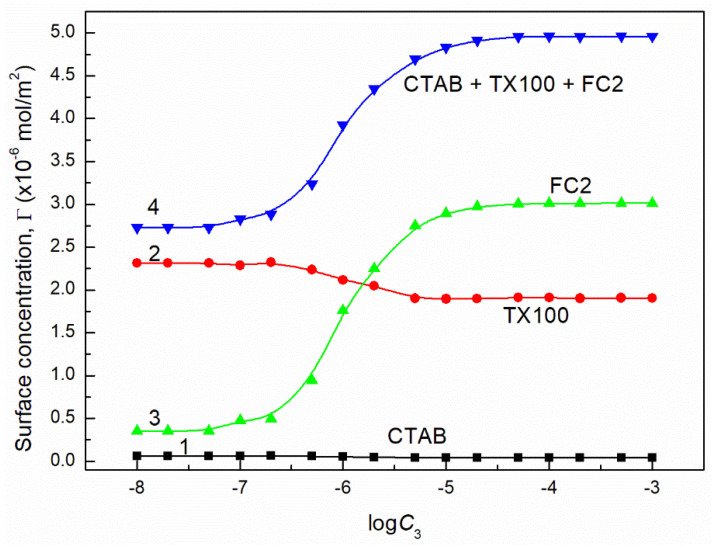
A plot of the concentration of CTAB (curve 1), TX100 (curve 2), FC2 (curve 3) and their sum (curve 4) in the mixed monolayer at the water-air interface formed by the CTAB + TX100 + FC1 mixture at the constant concentration of the binary CTAB + TX100 mixture ($C_{12}$) and $\gamma_{LV}$ = 60 mN/m as well as calculated from Equation (12) based on the contribution of the given surfactant to the reduction of water surface tension vs. the logarithm of the FC2 concentration ($logC_{3}$).

**Figure 18:**
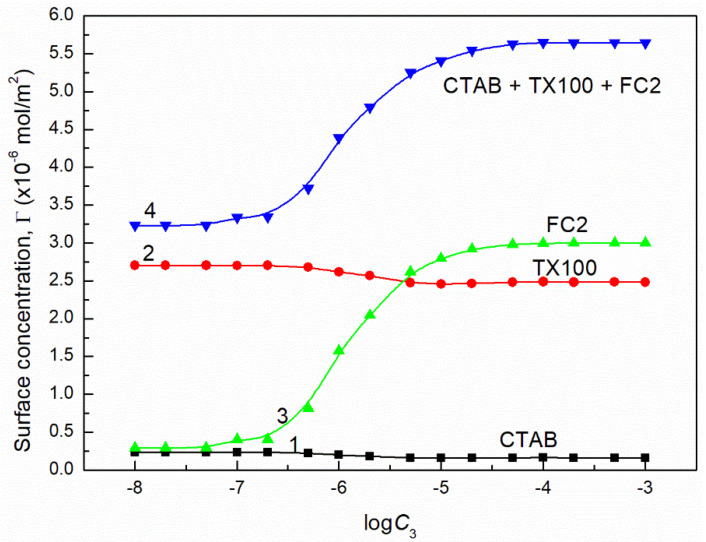
A plot of the concentration of CTAB (curve 1), TX100 (curve 2), FC2 (curve 3) and their sum (curve 4) in the mixed monolayer at the water−air interface formed by the CTAB + TX100 + FC1 mixture at the constant concentration of the binary CTAB + TX100 mixture ($C_{12}$) and $\gamma_{LV}$ = 50 mN/m as well as calculated from Equation (12) based on the contribution of the given surfactant to the reduction of water surface tension vs. the logarithm of the FC2 concentration ($logC_{3}$).

As was discussed above, the fraction of the contribution of the particular surfactant of the ternary mixture in the monolayer can be determined based on the monolayer pressure of all surfactants taken from their individual aqueous solutions. Thus, it can be written that:

(14) $$\pi_{1}=\pi X_{i}=\;\left(\gamma_{W}-\gamma_{LV}\right)X_{i},$$

where: $\gamma_{W}$ is the water surface tension. Introducing the $\pi_{i}$ values into Equation (13) the surface concentrations of CTAB, TX100, FC1 and FC2 were calculated and they are presented in Figure 13, Figure 14, Figure 15, Figure 16, Figure 17 and Figure 18.

From the calculations of the CTAB, TX100 and FC concentration in the monolayer at the water-air interface, it results that the increase of FC concentration at the constant concentration of CTAB and TX100 mixture results in a significant decrease of CTAB concentration but smaller decrease of TX100. However, the FC adsorption causes not only removal of CTAB and TX100 molecules but also an increase in the mixed monolayer density. This is confirmed by the increase of the sum of CTAB + TX100 and FC concentrations in the monolayer in comparison to that of the CTAB and TX100 mixture in the absence of FC surfactant. The maximal value of the sum of CTAB, TX100 and FC concentrations is greater than the Gibbs surface excess concentration of the single FC in the absence of CTAB and TX100 mixture at the water-air interface. As mentioned above this may result from the fact that during the adsorption of TX100 and FC not only dehydration of the tail of their molecules but also of the head takes place, due to strong interactions between these molecules as mentioned above.

### 2.4. Gibbs Free Energy of Adsorption

A measure of the tendency of the surfactant towards adsorption at the interface is the standard Gibbs free energy of adsorption ($\Delta G_{ads}^{0}$). The literature reports many methods which can be used for its determination [1,24,30].

If the Gibbs surface excess concentration of a given surfactant is known in the whole concentration range in the bulk phase, the Langmuir methods can be used [1,24]. These methods are based on the Langmuir equation modified by de Boer [32] and the linear one (Equation (11)) [1,24]. The Langmuir and de Boer equation has the form [1,24,32]:

(15) $$\frac{A_{0}}{A-A_{0}}exp\frac{A_{0}}{A-A_{0}}=\frac{C_{i}}{\varpi }exp\left(\frac{-\Delta G_{ads}^{0}}{RT}\right),$$

where: *A* is the area occupied by one molecule of the surfactant, *A*_0_ is the limiting area occupied by one molecule and ϖ is the number of the water moles in 1 dm^3^. The constant *a* can be determined from the linear Langmuir equation (Equation (16)). This constant fulfils the expression:

(16) $$a=\varpi exp\frac{\Delta G_{ads}^{0}}{RT},$$

The constant a can be determined also from the linear form of the Gu and Zhu equation and the Szyszkowski one (Equations (1) and (11)). There is also the relationship between the CMC and $\Delta G_{ads}^{0}$ described by the equation in the form [33]:

(17) $$\Delta G_{ads}^{0}=RTln\frac{CMC}{\omega }-\;\frac{\Delta \pi }{\Gamma_{i}^{max}},$$

where: Δπ is the difference between the water surface tension and the aqueous solution of the surfactant ternary mixture. From this equation the average values of $\Delta G_{ads}^{0}$ for the ternary mixture of surfactants can be calculated. However, from the above mentioned equations only $\Delta G_{ads}^{0}$ of FC can be determined (Table 3). As follows from Table 3 the $\Delta G_{ads}^{0}$ values of FC do not depend on the method used for their determination and are close to the FC1 and FC2 standard Gibbs fee energy of adsorption in the absence of CTAB and TX100 [7]. The absolute values of the $\Delta G_{ads}^{0}$ calculated from Equation (17) differ significantly from those determined by the Langmuir, Gu and Zhu as well as Szyszkowski methods. It is possible that this difference is connected with the Gibbs free energy of mixing of the surfactants in the monolayer at the water−air interface. If so, then the standard Gibbs free energy of CTAB, TX 100 and FC can be expressed in the form:

(18) $$\Delta G_{ads}^{mix}\;=\;X_{1}{\Delta G}_{ads}^{CTAB}\;+\;X_{2}\Delta G_{ads}^{TX100}\;+\;X_{3}\Delta G_{ads}^{FC}\;+\;G_{mix}^{E},$$

Taking into account the values of the standard Gibbs free energy of adsorption of the particular components of the ternary mixture of surfactants taken from the literature [9] and those of the Gibbs free energy of mixing determined based on Equation (7), the $\Delta G_{ads}^{mix}$ values were calculated from Equation (18). It proves that the $\Delta G_{ads}^{mix}$ values are close to those determined from Equation (17). This fact confirms the conclusions that the average standard Gibbs free energy of adsorption of the ternary mixture includes the Gibbs free energy of mixing. This may be a reason for the synergetic effect of the water surface tension reduction by the ternary mixture of the surfactants.

## 3. Materials and Methods

Hexadecyltrimethylammonium bromide (CTAB) (Sigma-Aldrich, St. Louis, USA), p-(1,1,3,3-tetramethylbutyl) phenoxypoly (ethylene glycol) (Triton X-100, TX100) (Sigma-Aldrich), Zonyl FSN-100 (CF6EO14, FC1) (DuPont, Wilmington, Delaware, USA) and Zonyl FSO-100 (FC5EO10, FC2) (DuPont) (100% nonionic fluorocarbon surfactants) were used without any further purification. FC1 and FC2 are ethoxylated nonionic fluorosurfactants, having an average 14 (from 1 to 26) and 10 (from 1 to 16) oxyethylene units and, 6 (from 1 to 9) and 5 (from 1 to 7) CF_2_ groups, respectively. There were studied the following ternary mixtures of surfactants:

m1 (60)—CTAB + TX100 (*α* CTAB = 0.2, *𝛾_LV_* = 60 mN/m, *C*_12_ = 4 × 10^−6^ M) + FC1 (*C*_3_ = 10^−8^–10^−3^ M)

m1 (50)—CTAB + TX100 (*α* CTAB = 0.2, *𝛾_LV_* = 50 mN/m, *C*_12_ = 2.4 × 10^−5^ M) + FC1 (*C*_3_ = 10^−8^–10^−3^ M)

m2 (60)—CTAB + TX100 (*α* CTAB = 0.2, *𝛾_LV_* = 60 mN/m, *C*_12_ = 4 × 10^−6^ M) + FC2 (*C*_3_ = 10^−8^–10^−3^ M)

m2 (50)—CTAB + TX100 (*α* CTAB = 0.2, *𝛾_LV_* = 50 mN/m, *C*_12_ = 2.4 × 10^−5^ M) + FC2 (*C*_3_ = 10^−8^–10^−3^ M)

Thus, for example, the mixture m1 (60) was prepared by adding FC1 at different concentrations to the binary mixture of CTAB + TX100 where the mole fraction of CTAB in the bulk phase, $\alpha_{1}$ was equal to 0.2 at $\gamma_{LV}$ of the binary mixture equal to 60 mN/m and the concentration of the mixture ($C_{12}$) corresponding to the saturated mixed monolayer at the water-air interface at which the synergetic effect occurred in the reduction of the water surface tension [17]. The aqueous solutions of ternary mixtures were prepared using doubly distilled and deionized water obtained from a Destamat Bi18E distiller.

The surface tension ($\gamma_{LV}$) measurements of the aqueous solution of the above mentioned ternary surfactants mixtures were made at 293 K using the Krüss K100 tensiometer which was calibrated before the measurements, based on water and methanol. If the water surface tension was equal to 72.8 mN/m and methanol to 22.5 mN/m, then it was assumed that the measured values of $\gamma_{LV}$ should be correct. The surface tension measurements procedure was described in detail earlier [7]. For each concentration of the aqueous solution of ternary mixtures, at least 10 measurements were made. The standard deviation was ± 0.1 mN/m and the uncertainty of the surface tension measurements was equal from 0.3% to 0.9%.

## 4. Conclusions

On the basis of the obtained results and their analysis it is possible to state that:

The dependence between the surface tension of the aqueous solution of the ternary mixtures, including CTAB, TX100 and FC1 or FC2 and the concentration of FC at the constant CTAB and TX100 mixture concentrations, can be described by the Szyszkowski equation only at the constant CTAB and TX100 mixture concentration at the surface tension of its aqueous solution equal to 60 mN/m in the range of FC surfactant concentration from zero to CMC. However, it is possible to describe this dependence by the exponential function in the whole range of FC concentrations.

One can predict the isotherm of the surface tension of the aqueous solution of a CTAB, TX100 and FC1 or FC2 mixture for the system in which the concentration of the binary TX100 and CTAB mixture is constant and the FC variable, using the Fainerman and Miller equation, if the most proper values of the area occupied by one mole of the mixture can be established in this equation. It was found that this area could be determined based on the molar area of single surfactants of the mixture and their ratio in the contribution to the water surface tension reduction. This ratio can be established based on the monolayer pressure of the ternary mixture components in their single aqueous solution at the same concentration as in the mixture.

The sum of the ratios for CTAB and TX100 and that of FC is close to the molar ratio calculated using the Rosen and Hua theory. Based on these ratios it was possible to determine the contribution of the particular components of the ternary mixture to the reduction of the water surface tension and to determine their concentration in the monolayer at the water−air interface using the Frumkin equation.

With the addition of FC to a binary mixture of CTAB and TX100 the density and amount of adsorbed molecules increase. However, the FC forces out the CTAB molecules largely and TX100 ones insignificantly.

The maximal concentration of the CTAB, TX100 and FC1 or FC2 mixture in the monolayer at the water−air interface is higher than that of FC in the absence of CTAB and TX100.

The standard Gibbs free energy of adsorption of the ternary mixture of surfactants at the water−air interface can be predicted based on the standard Gibbs free energy of adsorption of the components of the mixture and Gibbs free energy of surfactants mixing. This energy is related to the synergetic effect of the surfactants mixture in the reduction of water surface tension.

## Figures and Tables

**Table 1:** The values of the constants of the second order exponential function describing the relationship between the surface tension and the fluorocarbon surfactant concentration (Equation (2)) of CTAB + TX100 + FC1 (m1 (60), m1 (50)) and CTAB + TX100 + FC2 (m2 (60), m2 (50)) mixtures at CTAB + TX100 $\gamma_{LV}$ equal to 60 and 50 mN/m, respectively.

Mixture	$A_{1}$	$A_{2}$	$t_{1}$	$t_{2}$	$y_{0}$
m1 (60)	17.39734	16.53115	3.75681 × 10^−6^	2.84267 × 10^−5^	26.00475
m1 (50)	18.94709	4.93928	1.10939 × 10^−5^	1.10626 × 10^−4^	26.14665
m2 (60)	15.37929	24.31508	5.25560 × 10^−6^	3.47902 × 10^−5^	20.52881
m2 (50)	7.45181	22.35440	7.41333 × 10^−5^	1.68900 × 10^−5^	20.30326

**Table 2:** The values of the parameters of intermolecular interaction, $\beta^{\sigma }$, (Equation (6)) activity coefficients of CTAB + TX100 mixture, $f_{12}$, and fluorocarbon surfactant, $f_{3}$ (Equations (8) and (9)) and Gibbs excess free energy of mixing, $G_{mix}^{E}$ (Equation (7)) of CTAB + TX100 + FC1 (m1 (60), m1 (50)) and CTAB + TX100 + FC2 (m2 (60), m2 (50)) mixtures at CTAB + TX100 $\gamma_{LV}$ equal to 60 and 50 mN/m, respectively, at the different fluorocarbon surfactants’ surface tension.

Mixture	$\gamma_{LV}\;(\mathrm{mN}/m)$	$\beta^{\sigma }$	$f_{12}$	$f_{3}$	$G_{mix}^{E}\;(\mathrm{kJ}/\mathrm{mol})$
m1 (60)	55	−7.01610	0.15843	0.18866	−4.27016
	50	−5.21987	0.16282	0.41528	−3.07662
	45	−4.94660	0.11637	0.56414	−2.70309
	40	−4.66777	0.08598	0.70261	−2.26693
m1 (50)	47.5	−12.45280	0.06581	0.02925	−7.55158
	45	−9.73223	0.09052	0.08508	−5.92669
	42.5	−7.68936	0.11361	0.18539	−4.66383
	40	−6.53262	0.12019	0.29799	−3.90151
m2 (60)	55	−6.31109	0.209705	0.20320	−3.84336
	50	−4.85452	0.19322	0.42806	−2.87703
	45	−4.45539	0.13754	0.61067	−2.40961
	40	−4.27784	0.09733	0.74547	−2.01515
m2 (50)	47.5	−11.34600	0.08949	0.03712	−6.86813
	45	−9.29972	0.10832	0.08809	−5.66073
	42.5	−7.79572	0.12054	0.16714	−4.73926
	40	−6.79500	0.12230	0.26211	−4.08609

**Table 3:** The values of the standard Gibbs free energy of adsorption (kJ/mol) of FC1 and FC2 (Equations (15) and (16)) and this energy for the mixtures (Equations (17) and (18)).

Equation	m1 (60)	m1 (50)	m2 (60)	m2 (50)
Equation (15)	−42.08	−38.27	−41.24	−38.75
Equation (16)	−44.57	−41.65	−42.77	−40.36
Equation (16)	−46.57	−45.79	−44.78	−43.64
Equation (16)	−44.64	−38.42	−42.44	−43.64
Equation (17)	−48.04	−48.72	−45.62	−46.67
Equation (18)	−47.92	−48.30	−45.93	−47.55

## Data Availability

Not applicable.

## Sample Availability

Samples of the compounds are not available from the authors.
